# Alternative and Reciprocal Training

**Published:** 1921-05-14

**Authors:** 


### NURSING AUTHORITIES IN CONFERENCE.

### Alternative and Reciprocal Training.

The informal Conference convened by the General Nursing Council for England and Wales on Thursday, April 28, at the rooms of the Royal Society of Medicine, having discussed in the morning the draft syllabus (reported in The Hospital of May 7), reassembled in the afternoon to consider "The Supplementary Parts of the Register in conjunction with Alternative and Reciprocal Training." Mr. J. C. Priestley, K.C., Chairman of the General Nursing Council, again occupied the chair.

### The General-Hospital Point of View.

Miss Sparshott, C.B.E., R.R.C., read the opening paper. Her suggestions, she said, were only in outline, in order to start discussion. She put forward five systems of training to qualify nurses to enter for the examination of 1924: (1) Three years in a recognised general hospital, with a resident medical officer, on the syllabus of the General Nursing Council; (2) conjoint training through a "mother hospital" and three or four small hospitals attached, able to do the teaching required by the syllabus, one of these to be a small Poor-law infirmary giving the needed medical work; (3) training for two years in a fever or children's hospital and two years in a general hospital; (4) a three-year general-hospital training, with a fourth year for public health, child welfare, district nursing, midwifery, tuberculosis, fever, venereal diseases, one or more subjects; (5) a small general hospital affiliated to a women's and children's, and, if possible, to an eye hospital and ear and throat hospital. Four years' training would be required for all these alternative systems except (1). For the purpose of adequate and efficient grouping a geographical survey by the hospital authorities, with suggestions for grouping, would be helpful to the General Nursing Council.

### The Supplementary Register for Mental Nurses.

Dr. Bedford Pierce, M.D., said that, although mental nurses are only represented on the General Nursing Council by two persons, one only of whom is a nurse, there is no lack of sympathy shown by the Council with the mental nurse, to whose circumstances the utmost consideration is shown. Mental nursing is in some respects better placed than general nursing, and already has a one-portal system. The M.-P.A. certificate is recognised in every mental hospital, and it is desirable in the future to build on the foundation laid by the M.-P.A. to avoid break in the continuity of training and examinations. He desires to see more connection than at present between mental and general nursing, with ample facilities for experience in different branches of nursing.

### Poor-Law Hospitals.

In the absence, owing to serious illness, of Miss Seymour Yapp, the Chairman read her paper. On Miss Cox-Davies' motion, seconded by Miss Dowbiggin, the Conference passed a vote of sympathy with Miss Yapp in her illness; her absence, said Miss Dowbiggin, was a great loss to the meeting. Miss Yapp, in considering the position generally, divided Poor-law hospitals into three classes: (1) Minor training-schools, without a resident medical officer, at present granting three-year certificates; (2) recognised training-schools of medium size, from 250 to 500 beds; (3) complete major training-schools—*i.e.*, hospitals with from 500 to 1,500 beds. The schools in (1), she suggested, might become preliminary schools, taking girls of nineteen preferably for two years only, and working on the first year's syllabus; after passing an examination on this, the nurses should enter the complete major school (3) and take two years' further training, ranking as second- and third-year nurses; the latter class of school should reserve up to one-third of their vacancies for girls from minor schools. The medium-size schools (2), where surgical-teaching material is lacking, might affiliate with (*a*) special hospitals (preferably women's and children's), (*b*) small general or cottage hospitals. The nurses, after a two-year course on the first year's syllabus in hospitals (*a*) or (*b*), should enter the medium-size school (2) for another two years, ranking as second-year nurses, and should be eligible for the State examination; or (*c*) the local general hospital for a two or six months' course, as may be necessary, taken after the eighteenth month in the medium school. This is done at the Lake Hospital with satisfactory results, and this hospital has also taken probationers with two years' training from small schools. The experiment was a success.

### Fever Nurses.

Miss S. A. Villiers' paper dealt with the advantages and disadvantages attaching respectively to fever training (*a*) before or (*b*) after general training. The advantages to fever hospitals of (*a*) include a larger available supply of probationers, whilst in (*b*) better nursing would be secured and there would be less infectious illness among the nurses. But (*b*), with its shorter training in the fever hospital (one year after general training as against two years before general training, in (*a*)), would be more expensive. Miss Villiers thinks if (*b*) were adopted the provision of assistance by ward orderlies or nursing attendants might be necessary.

### Hospitals for Children.

Miss Coulton read a paper on reciprocal training in connection with general hospitals for adults and general hospitals for children, the main theme of which was the importance of a three years' training in a hospital for children for the nurse who intends to specialise in their nursing, and of a three years' training in a general hospital in addition for a sister in a children's hospital or in charge of a ward for children in a general hospital. Miss Coulton points out clearly the advantages of training in a children's hospital preceding that in the general hospital, among which is the earlier age at which a probationer is accepted at the former, thus tending to increase the supply of nurses.

### Will the Supplementary Registers Continue?

Dr. Goodall's short paper on this subject recited the various supplementary Registers laid down in the Act—*i.e.*, for mental, male, and children's nurses. In addition, the General Nursing Council has set up the Fever Nurses' Supplementary Register. He suggested that the mental and male nurses' Registers will be permanent, as they provide for a class who can never get on the General Register, but that those for children's and fever nurses would in course of time be really supplementary registers—*i.e.*, contain the names of nurses who have supplemented their general by a special training, and that these Registers may come to be disused if training in children's and infectious diseases in future forms part of the course of training of every general-trained nurse.

### Views and Questions.

Miss Beatrice Kent (President of the Professional Union of Trained Nurses) was the first speaker in the discussion. She urged at length the necessity for instruction in public health nursing and for the provision of trained nurses in prisons. Miss Tisdale (Hospital for Sick Children, Great Ormond Street) endorsed Miss Coulton's views on the training of nurses in children's hospitals, and expressed the hope that there will always be a Supplementary Register for Children's Nurses, for many nurses do not wish to take general training. She asked matrons of general hospitals to consider admitting trained children's nurses as second-year probationers. Miss Barton, R.R.C. (Matron, Chelsea Poor-law Infirmary), associated Poor-law matrons with the message of sympathy to Miss Yapp. She said the minor schools are anxious to remain as training-schools, having been so recognised by the Ministry of Health. Counting by numbers is unsatisfactory; some of the best schools are perhaps those having only 300 to 400 beds. Miss Foster asked if each training-school would continue to give its own certificate; to which Mrs. Bedford Fenwick, at the Chairman's request, replied. She pointed out that such a certificate would give the nurse no legal status. Under the Nurses' Registration Act the examination of the General Nursing Council is the only one which can qualify for the Register. Miss Vergette (Royal Victoria Hospital, Dover) pleaded for help for the small hospitals. Dr. Foord Caiger (Medical Superintendent, South-Western Fever Hospital, and Treasurer, Fever Nurses' Association) congratulated Miss Villiers on her statement of the relative advantages and disadvantages of the two schemes of training. He said the fever nurse has never desired a supplementary register apart from general training. If it is contemplated to put on the Fever Nurses' Register nurses who have not had any general training, he hopes that that Register will not be largely used. General training is essential to a nurse if she is to be properly qualified. Miss Neville (Children's Hospital, Pendlebury) endorsed Miss Coulton's views and expressed the hope that there will always be a Supplementary Register for Children's Nurses; she was asked by Manchester doctors who specialise in children to say how much importance they attach to this special Register. Miss Ind (Stratford-on-Avon Hospital) gave figures of the large amount of work done in small hospitals. There are 333 hospitals of fifty beds and less, and forty-seven of fifty to 100 beds, and between them they employ 1,190 trained and 1,271 probationer nurses. Many recruits are lost to the nursing profession each year owing to the non-recognition of small hospitals. She suggested they should take probationers for two years, giving during that time the equivalent of the first year's training in a general training-school, and that the age-limit of the small hospital should be one year below that for the large training-school. Miss E. M. Musson, R.R.C. (General Hospital, Birmingham), spoke from practical experience of reciprocal training; the difficulty is in not having the first-year probationers, but she believes a uniform standard of training will remedy this. It would be an advantage if probationers at general hospitals could be allowed to go to special hospitals for a three months' course. Miss Rossiter, who trained first in a London fever hospital and afterwards at St. Bartholomew's Hospital, spoke of the value of fever training in doing general training. Miss Clearey (Mental Hospital, Norwich) asked what general training would be necessary for the mental-trained nurse with the M.-P.A. certificate to get on the General Register. Miss Bramwell (Eye and Ear Infirmary, Liverpool) asked the position of a probationer who had trained for two years in an eye and ear infirmary of seventy-two beds, to which the Chairman replied that it was doubtful whether she would have recognition of any kind. Miss Cann (Norfolk and Norwich Hospital) suggested the grouping by regional committees of suitable hospitals with uniform training. She confessed to an old-fashioned prejudice for choosing her own probationers. She would lower the standard of number of beds in a hospital to fifty, and let them join up with workhouse infirmaries so as to give experience in nursing chronic cases. Miss Croucher (Tynemouth Infirmary) suggested an exchange or interchange of staffs between infirmary and local hospital of under 100 beds. A travelling sister-tutor should not have more than two hospitals to teach. Miss C. E. Vincent, R.R.C. (Leicester Royal Infirmary), speaking from experience of reciprocal training, as she takes nurses from special hospitals for surgical work, finds that most nurses coming from other hospitals in their second year cannot be put to rank with the second-year nurse in the large general hospital. Miss Brodie (City Mental Hospital, Leicester) asked whether mental nursing will be considered by the General Nursing Council in regard to reciprocal training. The Chairman gave an affirmative answer. Miss Bushby (Queen's Hospital for Children) asked, if a candidate failed in the State examination, how many times she would be allowed to sit for it. The Chairman said that had not been considered. Miss Breay suggested that if individual hospitals continue to give their own certificate it should not be given till after the State examination had been passed, so as to guard against a favourable certificate being given to a nurse who might fail at the State examination. The Chairman read out a question whether there will be one standard of training laid down for fever hospitals as qualifying for entrance to a general training-school, and replied, "No doubt." Dr. Bedford Pierce, replying to Miss Clearey's question and to a similar one handed up by Miss Sinclair, said the question had not been considered by the Council as a whole; it is one of extreme difficulty. His own opinion is that if a nurse has been satisfactorily trained in mental nursing in a hospital of sufficient size approved by the Council, she should be excused one year of her hospital training if she wishes to come on the General Register. Miss Davies (Royal National Hospital, Ventnor) asked if training in chest nursing of girls of eighteen, staying two years and passing on to a general hospital, would be recognised if the syllabus is taken as ground work and worked through in the first year.

Mrs. Bedford Fenwick's Summing Up.

Mrs. Bedford Fenwick said the question of reciprocal and alternative training was forced upon them by the present conditions of medical treatment and science. Forty years ago a general hospital was more inclusive than it is now. Their aim must be to evolve schemes to utilise all the material available so as to do justice to all branches. Miss Sparshott had made some very able and simple suggestions. It will be impossible to continue on the present individual system, and grouping seems to be the most practicable for those hospitals which cannot be inclusive in their training. Nothing must be done to prevent the special hospitals and other groups from being able to participate in a scheme of alternative and reciprocal training, to which she presumed all were agreed. It is to be hoped that admission to training or to the wards of persons under the name of attendants will not be considered. American action in this connection is disastrous. Mrs. Fenwick expressed the hope that that day's Conference would be the first of many. In reply to Miss Bramwell's question, she imagines no nurse will be eligible without one year's general training, and in reply to Miss Elsie Mussett, whom the Chairman then invited to put her question, Mrs. Fenwick said she believes a hospital of 100 beds will certainly be recognised as an inclusive training-school if the work is good and acute. That was her opinion; the Council has not yet defined the number of beds to constitute a training-school for the General Register.

A vote of thanks was passed to the Royal Society of Medicine for the use of the rooms and one to the Chairman, Mr. Priestley. In proposing the latter, Miss Cox Davies said that without the devoted help given by him in the work of organising the nursing profession the General Nursing Council would be in a sorry plight.

